# Impact of multi-disciplinary treatment strategy on systolic heart failure outcome

**DOI:** 10.1186/s12872-019-1214-0

**Published:** 2019-10-15

**Authors:** Shyh-Ming Chen, Yen-Nan Fang, Lin-Yi Wang, Ming-Kung Wu, Po-Jui Wu, Tsung-Hsun Yang, Yung-Lung Chen, Chi-Ling Hang

**Affiliations:** 1 Section of Cardiology, Department of Internal Medicine, Kaohsiung Chang Gung Memorial Hospital and Chang Gung University College of Medicine, 123 Tai Pei Road, Niao Sung District, Kaohsiung City, 83301 Taiwan, Republic of China; 2 Heart Failure Center, Kaohsiung Chang Gung Memorial Hospital, 123 Tai Pei Road, Niao Sung District, Kaohsiung City, 83301 Taiwan, Republic of China; 3 Department of Physical Medicine and Rehabilitation, Kaohsiung Chang Gung Memorial Hospital and Chang Gung University College of Medicine, 123 Tai Pei Road, Niao Sung District, Kaohsiung City, 83301 Taiwan, Republic of China; 4 Department of Psychiatry, Kaohsiung Chang Gung Memorial Hospital and Chang Gung University College of Medicine, 123 Tai Pei Road, Niao Sung District, Kaohsiung City, 83301 Taiwan, Republic of China

**Keywords:** Heart failure, Disease management program, Readmission, Cardiac rehabilitation

## Abstract

**Background:**

Patients with reduced ejection fraction have high rates of mortality and readmission after hospitalization for heart failure. In Taiwan, heart failure disease management programs (HFDMPs) have proven effective for reducing readmissions for decompensated heart failure or other cardiovascular causes by up to 30%. However, the benefits of HFDMP in different populations of heart failure patients is unknown.

**Method:**

This observational cohort study compared mortality and readmission in heart failure patients who participated in an HFDMP (HFDMP group) and heart failure patients who received standard care (non-HFDMP group) over a 1-year follow-up period after discharge (December 2014 retrospectively registered). The components of the intervention program included a patient education program delivered by the lead nurse of the HFDMP; a cardiac rehabilitation program provided by a physical therapist; consultation with a dietician, and consultation and assessment by a psychologist. The patients were followed up for at least 1 year after discharge or until death. Patient characteristics and clinical demographic data were compared between the two groups. Cox proportional hazards regression analysis was performed to calculate hazard ratios (HRs) for death or recurrent events of hospitalization in the HFDMP group in comparison with the non-HFDMP group while controlling for covariates.

**Results:**

The two groups did not significantly differ in demographic characteristics. The risk of readmission was lower in the HFDMP group, but the difference was not statistically significant (HR = 0.36, *p* = 0.09). In patients with ischemic cardiomyopathy, the risk of readmission was significantly lower in the HFDMP group compared to the non-HFDMP group (HR = 0.13, *p* = 0.026). The total mortality rate did not have significant difference between this two groups.

**Conclusion:**

The HFDMP may be beneficial for reducing recurrent events of heart failure hospitalization, especially in patients with ischemic cardiomyopathy.

**Trial registration:**

Longitudinal case-control study ISRCTN98483065, 24/09/2019, retrospectively registered.

## Background

Heart failure (HF) is a growing epidemic worldwide, owing to the aging populations and the increasing survival of patients presenting with acute myocardial infarction and various other heart diseases [1]. The HF readmission rate and mortality rate are high in Taiwan. Taiwan Society of Cardiology-Heart Failure with reduced Ejection Fraction (TSOC-HFrEF) registry data reveal an HF readmission rate of 38.5% and an HF total mortality rate of 15.9% in 1 year after the index hospitalization [2]. During the 1-year period after index hospitalization, more than half (53.6%) of HF patients die, are hospitalized for HF, or require left a ventricular assistive device or heart transplantation [2]. Therefore, improvements in HF care in Taiwan are urgently needed. In Taiwan, the heart failure disease management programs (HFDMPs) led by cardiovascular nursing specialists have proven effective for decreasing adverse outcomes of HF and have achieved HF treatment cost savings of up to 41.8% [3]. These programs can decrease the rate of readmission for HF or other cardiovascular causes by up to 30% and have a trend toward lower mortality rate by a systemic meta-analysis [4]. According to recently published guidelines, a multidisciplinary team should provide for HF patients with class I level A evidence [5]. Despite convincing evidence of its effectiveness, however, HFDMPs are not been widely used in Taiwan. One reason is that the best design and implementation of an HFDMP is unclear. Additionally, some HFDMPs show not improvements in health status compared with standard care [6, 7]. Another question is whether HFDMP should be provided to all HF patients or only specific subsets.

Therefore, the objective of this study was to design a multi-disciplinary, multi-faceted HFDMP and to compare it with standard care in a population of HF patients with multiple co-morbidities. Patients hospitalized for HF with left ventricular ejection fraction (LVEF) < 40% were enrolled and studied over a 1-year follow-up period.

## Method

### Study design

This observational cohort study compared rates of mortality and cardiovascular readmission between an HFDMP group and a non-HFDMP group. The subjects included 159 patients admitted for systolic heart failure (LVEF < 40%) at a single medical center in south Taiwan from July, 2013 to December, 2014. Of these, 64 consecutive patients were enrolled in a non-HFDMP group that received standard care from July, 2013 to June, 2014, and 95 consecutive patients were enrolled in an HFDMP group that received the HFDMP intervention from May to December, 2014. The inclusion criteria were HF with reduced EF (EF < 40%), radiographic evidence of pulmonary congestion or typical symptoms and signs of HF, age > 18 years, and NYHA functional class II-IV. The exclusion criteria were severe respiratory failure under ventilator support, dementia, expectation of short survival, discharge to a geriatric clinic or home care, or current follow-up treatment at the nurse-led HF clinic.

The HFDMP comprised patient education delivered by the lead nurse, dietary consultation, psychological consultation and assessment, and a cardiac rehabilitation program provided by a physical therapist. The patients were followed up for at least 1 year or until death. The outcome measures were readmissions related to cardiovascular problems and all-cause mortality. Clinical demographic data, laboratory findings, and medications were used for risk adjustment.

### Intervention protocol

The education program included the following components:

- Explanation of HF and its causes
- Differences between expected and severe symptoms and how to monitor them
- Symptoms that often occur before HF hospitalization, i.e., dyspnea, edema, fatigue, cough, chest pain, sudden weight gain, difficulty breathing while sleeping, palpitations
- Purpose of each medication and strategies for maintaining compliance with the prescribed dosage
- Importance of risk factor modification
- Individualized recommendations for dietary restrictions on sodium, fluid, and alcohol
- Importance of recording body weight and any changes from a daily basis
- Recommendations for exercise and rest
- Recommended behavioral changes
- How to cope with the disease (psychosocial care)

Patients are also taught the following skills

- Recognition of symptoms
- Important signs and symptoms
- Timely response to symptoms
- When to call the health care provider
- How to differentiate between high- and low-sodium foods

The cardiovascular lead nurse contacted the patient by telephone within 3 days after discharge. An appointment at the outpatient clinic was arranged within 1 to 2 weeks after discharge. The purposes of the telephone call were to reinforce self-management and recognition of HF symptoms and to screen post-discharge health status.

The lead nurse arranged further consultations as needed with the pharmacist, dietician, educator, and psychologist. The patient was encouraged to contact the nurse directly if any questions or problems arose. All patients in the intervention group received phase I cardiac rehabilitation before discharge, and some also received phase II cardiac rehabilitation.

Management and follow up of patients in the control group were performed by the participating physicians according to current clinical practices. No standard format or guidelines were established for the education program provided by the nurse or for the consultations provided by the dietitian and psychologist. Patients in non-HFDMP group did not have cardiac rehabilitation program as patients in HFDMP did.

### Statistical analyses

The χ2 and t test were used to compare patient characteristics between the HFDMP and non-HFDMP groups. Since a hospital readmission for HF is a recurrent event, Cox proportional hazards regression analysis of recurrent events was performed using the Wei, Lin, and Weissfeld (1989) method based on marginal Cox models [8] to compare the hazard ratio (HR) of death or readmission between the HFDMP group and the non-HFDMP group while controlling for covariates. The interaction term under the previous model of recurrent events was also used to investigate whether ischemic cardiomyopathy differed between the two groups. This study was approved by the Human Investigation Committee of the Institutional Review Board of Chang-Gung Memorial Hospital in Kaohsiung, Taiwan.

## Results

Demographic and clinical characteristics of 159 heart failure patients are summarized in Table 1. The HFDMP and non-HFDMP groups did not significantly differ in age, gender, cardiovascular function, 1-year mortality, comorbidities, medications, or cardiac resynchronization therapy. However, the use of implantable cardioverter defibrillator (ICD) was significantly higher in the HFDMP group (12.63%) compared to the non-HFDMP group (1.56%). Covariate-adjusted analysis revealed that HF readmissions and mortality were lower in the HFDMP group. However, the difference in HF readmissions between the HFDMP group (29.67%) and the non-HFDMP group (30.51%) did not reach statistical significance (*p* = 0.91) (Table 2). Additionally, the difference in 1-year mortality between the HFDMP group (11.58%) and the non-HFDMP group (17.19%) did not reach statistical significance (*p* = 0.32). All patients in the HFDMP had received phase I cardiac rehabilitation. However, only 15 patients (15.8%) in the HFDMP group had received phase II cardiac rehabilitation. No patients in the non-HFDMP group had received cardiac rehabilitation.

**Table 1:** Demographic and clinical characteristics of 159 HF patients

Variables	HFDMP		non-HFDMP		*χ*^2^/*t*	*p*
	n/means	%/std.	n/means	%/std.		
Age	69.79	14.98	70.47	14.01	−0.29	.77
Gender					1.63	.20
Male	59	62.11	46	71.88		
Female	36	37.89	18	28.13		
peak VO_2_	14.07	3.32	17.50	2.12	−1.40	.18
VE/VCO_2_ at AT	39.92	6.46	33.00	0.00	1.47	.17
Mortality					1.01	.32
Survival	84	88.42	53	82.81		
Death	11	11.58	11	17.19		
LVEF	32.25	5.87	31.03	7.16	1.18	.24
Atrial Fibrillation					0.95^a^	.81
non AF	59	62.11	44	68.75		
Paroxysmal AF	24	25.26	14	21.88		
Persistent AF	3	3.16	1	1.56		
Permanent AF	9	9.47	5	7.81		
Ischemic CM	56	58.95	36	56.25	0.11	.74
Hypertension	74	77.89	50	78.13	0.00	.97
Diabetes mellitus	44	46.32	24	37.50	1.21	.27
Hyperlipidemia	56	58.95	30	46.88	2.24	.13
Stroke	23	24.21	13	20.31	0.33	.56
Old MI	31	32.63	23	35.94	0.19	.67
PAD	16	16.84	8	12.50	0.56	.45
CKD	59	62.11	44	68.75	0.74	.39
PCI	36	37.89	23	35.94	0.06	.80
Medication / Treatment						
ACEI/ARB	76	80.00	49	76.56	0.27	.60
β-blocker	62	65.26	40	62.50	0.13	.72
Aldactone	38	40.00	19	29.69	1.77	.18
Diuretics	74	77.89	48	75.00	0.18	.67
Digoxin	17	17.89	5	7.81	3.26	.07
ICD	12	12.63	1	1.56	6.24	.01
CRT	7	7.37	4	6.25	0.07^a^	1.00

*Abbreviations*: *HFDMP* heart failure disease management program, *VE* minute ventilation, *AT* anaerobic threshold, *LVEF* left ventricular ejection fraction, *AF* atrial fibrillation, *CM* cardiomyopathy, *HF* heart failure, *MI* myocardial infarction, *PAD* peripheral artery disease, *CKD* chronic kidney disease, *PCI* percutaneous coronary intervention, *ACEI* angiotensin converted enzyme inhibitor, *ARB* angiotensin receptor blocker, *ICD* implantable cardioverter defibrillator, *CRT* cardiac resynchronization therapy

^a^Fisher exact test

**Table 2:** Outcomes analysis

Variable	HFDMP (*n* = 95)	non-HFDMP (*n* = 64)	*P*-value
Readmission within 30 days (%)	4.30	3.28	1.00
Readmission within 6 months (%)	20.88	21.67	0.91
Readmission within 1 year (%)	29.67	30.51	0.91
Death within 30 days (%)	3.16	6.25	0.44
Death within 6 months (%)	10.53	12.50	0.70
Death within 1 year (%)	11.58	17.19	0.32

*Abbreviations*: *HFDMP* heart failure disease management program, *Non-HFDMP* standard care

**Fig. 1:**
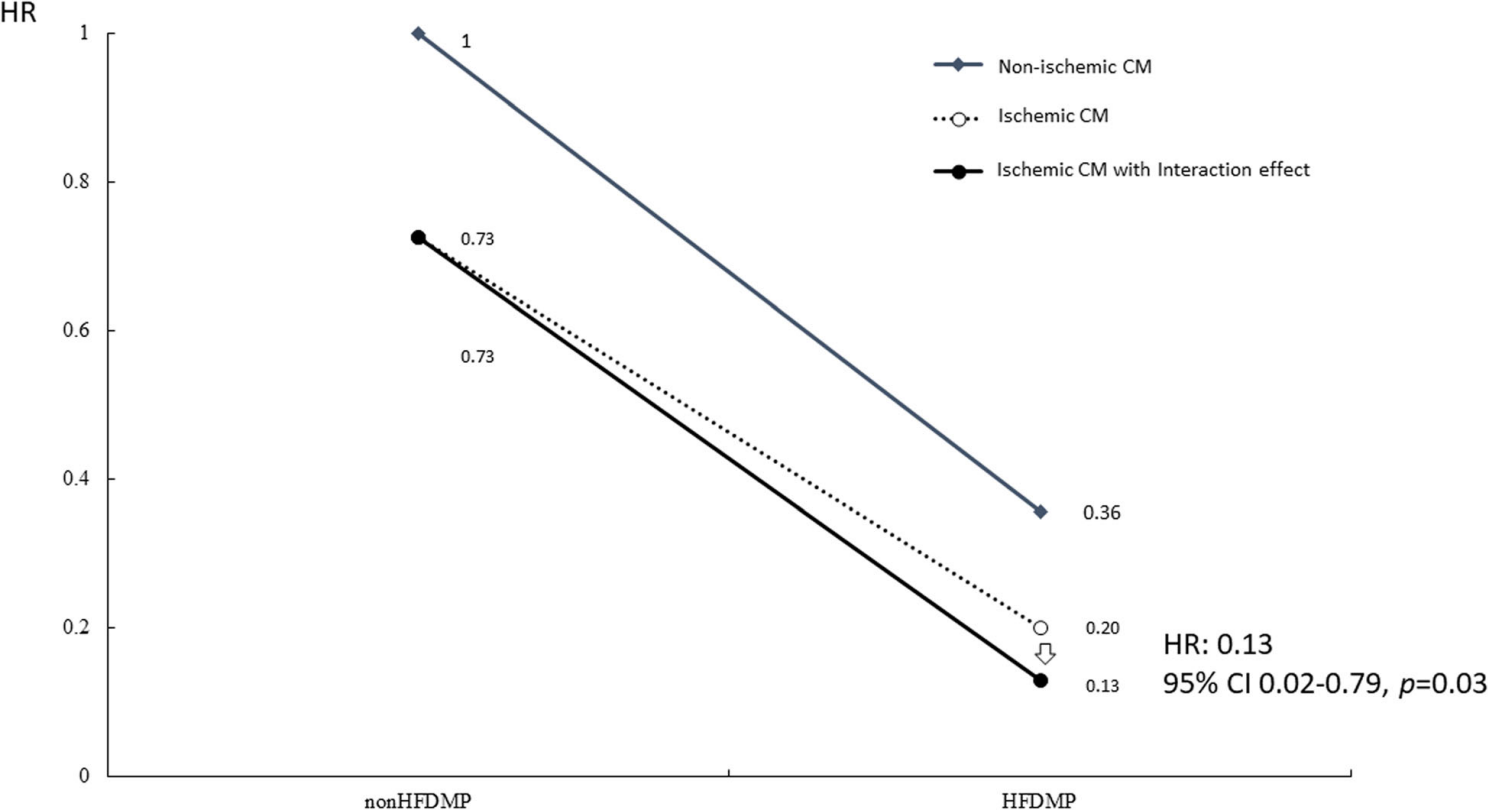
Cox model of recurrent events under heart failure disease management program (HFDMP). The Cox model of recurrent events showed that ischemic cardiomyopathy patients had a significantly lower risk of readmission compared to non-ischemic cardiomyopathy patients under HFDMP

Table 3 shows the Cox proportional hazard model results for recurrent events of cardiovascular hospitalization in the HF patients. Readmission risk was lower in the HFDMP group compared to the non-HFDMP group (HR = 0.36, 95% confidence interval [CI] 0.11–1.19, *p* = 0.09), but the difference did not reach statistical significance. The interacting terms in the previous Cox model of recurrence were further used to compare different subgroups of patients who had received HFDMP. Figure 1 shows that ischemic cardiomyopathy patients had a lower readmission risk compared to non-ischemic cardiomyopathy under HFDMP (HR = 0.13, 95% CI 0.02–0.79, *p* = 0.03).

**Table 3:** Results of Cox model of recurrent events of hospitalization in HF patients

Variables	*β*	*S.E.*	*HR*	*HR* 95% CI	*p*
Group					
non-HFDMP	ref.		1.00		
HFDMP	−1.03	0.62	0.36	0.11 – 1.19	.09
Age	0.03	0.02	1.03	0.99 – 1.06	.14
Gender					
Female	ref.		1.00		
Male	0.23	0.29	1.26	0.71 – 2.22	.43
Ischemic CM	−0.32	0.46	0.72	0.29 – 1.78	.48
Hypertension	−0.42	0.48	0.65	0.25 – 1.68	.38
Diabetes mellitus	−0.55	0.38	0.58	0.28 – 1.21	.14
Hyperlipidemia	0.00	0.68	1.00	0.27 – 3.77	1.00
Stroke	0.78	0.56	2.18	0.72 – 6.54	.17
Old MI	0.10	0.59	1.11	0.35 – 3.51	.87
PAD	0.51	0.52	1.67	0.60 – 4.67	.33
CKD	−0.78	0.52	0.46	0.17 – 1.26	.13
Atrial Fibrillation					
Without AF	ref.		1.00		
Paroxysmal	−0.30	0.61	0.74	0.22 – 2.47	.63
Persistent	−2.06	1.05	0.13	0.02 – 1.01	.05
Permanent	−0.32	0.79	0.73	0.15 – 3.42	.69
PCI	0.44	0.64	1.55	0.44 – 5.39	.49
LVEF	0.01	0.03	1.01	0.96 – 1.06	.73
Medication					
ACEI/ARB	0.13	0.58	1.14	0.37 – 3.54	.82
β-blocker	−1.70	0.44	0.18	0.08 – 0.43	<.01
Aldactone	0.98	0.54	2.68	0.93 – 7.68	.07
Diuretics	−0.79	0.54	0.45	0.16 – 1.29	.14
Digoxin	0.22	0.47	1.25	0.50 – 3.10	.64
ICD	0.96	0.61	2.60	0.78 – 8.63	.12
CRT	−1.15	1.37	0.32	0.02 – 4.65	

*Abbreviations*: *HFDMP* heart failure disease management program, *CM* cardiomyopathy, *HF* heart failure, *MI* myocardial infarction, *PAD* peripheral artery disease, *CKD* chronic kidney disease, *AF* atrial fibrillation, *PCI* percutaneous coronary intervention, *LVEF* left ventricular ejection fraction, *ACEI* angiotensin converted enzyme inhibitor, *ARB* angiotensin receptor blocker, *ICD* implantable cardioverter defibrillator, *CRT* cardiac resynchronization therapy, *HR* Hazard ratio, *CI* confidence interval

## Discussion

This observational cohort study revealed that HFDMP did not significantly reduce 1-year cardiovascular readmissions in HFrEF patients. However, the Cox model of recurrent events showed that ischemic cardiomyopathy patients had a significantly lower risk of readmission compared to all other subgroups of HFrEF patients. (HR = 0.13, 95% CI 0.02–0.79, *p* = 0.03). Disease management interventions for HF are highly heterogenous and complex and often yield mixed results in different HF populations [9–17]. Our intervention program was led by a cardiovascular nursing specialist and an HF cardiologist. The program also included cardiac rehabilitation and psychiatric interventions. Notably, progressive enlargement, dilatation, and global or regional dysfunction of the left ventricle can result from secondary myocardial damage in HF patients with ischemic cardiomyopathy [18, 19]. Myocardial remodeling may precede deterioration of exercise capacity and HF hospitalization in these patients [20]. Our disease management program included cardiac rehabilitation, which can reportedly improve cardiopulmonary function and reduce recurrent events of hospitalization in HF patients with ischemic cardiomyopathy [21].

Researchers and policy makers have shown great interest in the concept of preventing readmissions in HF patients. The 30-day readmission rate, which is an important measure of hospital performance, has been linked to financial penalties in the USA [22]. Additionally, 30-day readmission is associated with poor prognosis at 6-month follow up [23]. An analysis of 43,143 patients treated at 171 centers revealed that the hospitals with high risk-adjusted 30-day readmission rate also had higher 1-year all cause readmission rate (59.1% vs. 54.7%, respectively; *p* = 0.01) [24]. The HFDMP group in our study had a lower 1-year readmission rate (29.67%) compared to HF patients treated with standard care as reported in the TSOC-HFrEF registry (38.5%) [2] and in HF registries in other countries (e.g., 30.1% in IN-HF outcome registry in India [25] and 36% in registry in Saudi Arabia [26]). In comparison, the EHFS-2 registry in Europe reveals a 1-year morality rate of 21.9% for patients hospitalized with acute HF [27], and the IN-HF registry in India reveals a 1-year morality rate of 24.4% [25]. In a population-based cohort study from the United Kingdom 2000–2017, the overall one-year mortality decreased by 6.6% for people with a new diagnosis of heart failure from 25.8% in 2000 to 19.2% in 2016 [28]. In Asia, 1-year HF mortality rates are 8.9, 9.2 and 19.5% according to registry data for Japan (JCARE-CARD), Korea (KorHF), and Hong Kong (Hong-Kong HF), respectively. In comparison, the HFDMP group in our study had a 1-year mortality rate of 11.58%.

The HF nursing specialist has a key role as a case manager or coordinator of the HFDMP [29]. A competent HF nursing specialist is essential for providing the education and psychosocial interventions needed to improve drug compliance [30]. A large retrospective cohort study reported that noncompliance with drug therapy is associated with an increased risk of all-cause mortality and cardiovascular hospitalization [31]. In Taiwan, a descriptive, cross-sectional study revealed low self-care maintenance and management in HF patients [32]. A study of a Chinese HF patients further showed that an HFDMP led by a HF nursing specialist can reduce cardiovascular hospitalization and can substantially reduce hospital costs [33]. The results of our study are consistent with previous studies of HFDMPs that included additional components such as cardiac rehabilitation and psychosocial surveillance.

The aims of this study were focused on adverse outcomes. Therefore, we don’t have cost analysis for this study. We agree it’s an important issue for HFDM program and the results will provide helpful information for policy decision marker. In the believe that disease management program would be more cost-effective by decreasing HF readmission rate, Taiwan national health insurance (NHI) launched HF post-acute care program with multi-discipline team approach on July 1, 2017 [34].

This study has several limitations. First, this was a retrospective cohort study performed in a tertiary referral center in Taiwan. The study population comprised patients with LVEF < 40% at their first hospitalization for HF. Therefore, the results may not be generalizable to the spectrum of HF patients. Second, the patient number was small. Although this study included a control group, larger multicenter studies are needed for a clearer picture of the effectiveness of the HFDMP and a different way to present them. Third, although the HFDMP included psychosocial surveillance, no data were collected for the non-HFDMP group. Further prospective randomized studies are needed to determine the psychosocial effects of the HFDMP. Finally, the patient number differed between the intervention group and the control group. Nevertheless, except for ICD, baseline demographic and clinical characteristics did not significantly differ between the two groups.

## Conclusions

This study developed and evaluated the effectiveness of an HFDMP in de novo HF patients with LVEF < 40% over a 1-year follow-up period. Comparisons with the non-HFDMP group showed that the program reduced recurrent events of hospitalization, especially in HF patients with ischemic cardiomyopathy. Further studies are needed to investigate whether the difference resulted from the cardiac rehabilitation and psychosocial intervention received by the HFDMP group.

## Data Availability

The datasets generated and/or analyzed during the current study are available in the repository 10.6084/m9.figshare.9907250.

## Abbreviations

ACEI: Angiotensin converted enzyme inhibitor
AF: Atrial fibrillation
ARB: Angiotensin receptor blocker
AT: Anaerobic threshold
CKD: Chronic kidney disease
CRT: Cardiac resynchronization therapy
HF: Heart failure
HFDMP: Heart failure disease management program
HFrEF: Heart failure with reduced ejection fraction
ICD: Implantable cardioverter defibrillator
ICM: Ischemic cardiomyopathy
LVEF: Left ventricular ejection fraction
MI: Myocardial infarction
PAD: Peripheral artery disease
PCI: Percutaneous coronary intervention
VE: Minute ventilation
